# Implementation of a DVH Registry to provide constraints and continuous quality monitoring for pediatric CSI treatment planning

**DOI:** 10.1002/acm2.13131

**Published:** 2020-12-14

**Authors:** Esteban Sepulveda, Haley Patrick, Carolyn R. Freeman, John Kildea

**Affiliations:** ^1^ Medical Physics Unit Department of Physics McGill University Montréal Canada; ^2^ Division of Radiation Oncology Department of Oncology McGill University Montréal Canada

**Keywords:** biostatistics, craniospinal irradiation, dose–volume histogram

## Abstract

Craniospinal irradiation (CSI) is a complex radiation therapy technique that is used for patients, often children and teenagers/young adults, with tumors that have a propensity to spread throughout the central nervous system such as medulloblastoma. CSI is associated with important long‐term side effects, the risk of which may be affected by numerous factors including radiation modality and technique. Lack of standardization for a technique that is used even in larger radiation oncology departments only a few times each year may be one such factor and the current ad hoc manner of planning new CSI patients may be greatly improved by implementing a dose–volume histogram registry (DVHR) to use previous patient data to facilitate prospective constraint guidance for organs at risk. In this work, we implemented a DVHR and used it to provide standardized constraints for CSI planning. Mann–Whitney U tests and mean differences at 95% confidence intervals were used to compare two cohorts (pre‐ and post‐DVHR intervention) at specific dosimetric points to determine if observed improvements in standardization were statistically significant. Through this approach, we have shown that the implementation of dosimetric constraints based on DVHR‐derived data helped improve the standardization of pediatric CSI planning at our center. The DVHR also provided guidance for a change in CSI technique, helping to achieve practice standardization across TomoTherapy and IMRT.

## 1 INTRODUCTION

Approximately half of all cancer patients receive radiation therapy at some point over the course of their illness.^1^ Craniospinal irradiation (CSI) is the main treatment procedure for central nervous system tumors, most typically medulloblastoma.^2^, ^3^ This specific malignancy accounts for nearly one‐quarter of all pediatric central nervous system neoplasms.^4^, ^5^ Compared to other CNS tumors, medulloblastoma is significantly more challenging to treat due to the high probability (40% of cases) to metastasize through the craniospinal fluid of the neural axis.^5^, ^6^ Because of this, patients with medulloblastoma receive postoperative CSI covering the whole brain and the spinal axis.^2^, ^5^, ^7^

Since many organs at risk (OARs) lie adjacent or close to the neural axis target in CSI, the risk of long‐term toxicities is an important consideration.^8^, ^9^, ^10^ The frequency and severity of late effects depend on the age of the patient^11^ but overall 60% to 90% of patients develop chronic side effects.^12^, ^13^, ^14^, ^15^

Due to the lack of published organ tolerances for CSI planning, physicians and dosimetrists regularly refer to plans of previously treated patients for guidance. Although the Pediatric Normal Tissue Effects in the Clinic (PENTEC^11^, ^16^) consortium has recently begun a concerted effort to determine organ dose tolerances for pediatric patients, recommendations similar to those provided for adults by the Quantitative Analysis of Normal Tissue Effects in the Clinic (QUANTEC^17^, ^18^) effort may still be several years away. In the quality improvement project we describe here, we developed a dose–volume histogram registry (DVHR) to review historical dosimetric data from CSI treatment plans and used it to derive institutional dosimetric constraints for OARs. We hypothesized that the implementation and use of our DVHR would provide us with planning constraints and enable continuous quality monitoring of pediatric CSI treatment planning at our institution.

## 2 MATERIALS AND METHODS

We created our DVHR in 2014 to enable the visualization and comparison of DVHs from multiple patients simultaneously. A web interface allows for interactive visualization of DVHs using a dynamic JavaScript charting library (Highcharts, Vik i Sogn, Norway). The DVHR was developed using Django, an open‐source web framework based on Python, allowing the use of libraries for data analysis such as pandas, lifelines, and scipy.^19^, ^20^ The DVHR front‐end uses HTML, CSS, and JavaScript in order to provide an interactive web‐based user interface. The Python backend interfaces with a MySQL database.

**FIG. 1:**
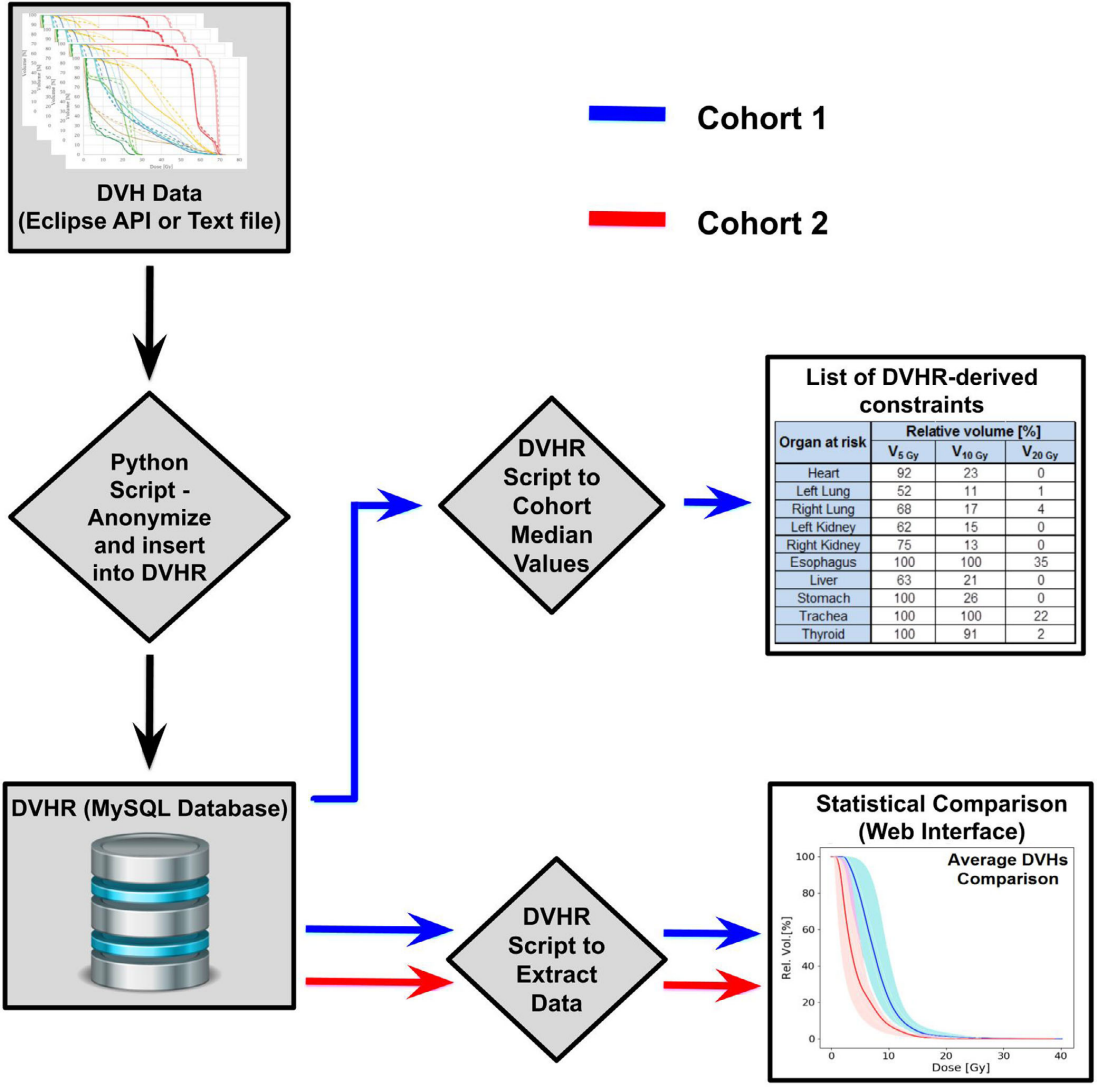
Schematic showing the data flow of the DVH registry. Using a Python Script, DVH data from all patients meeting the selection criteria in the Eclipse treatment planning system were accessed via the Eclipse API, anonymized, filtered, renamed, and inserted into the DVHR. The internal serial number of the patient within the treatment planning system was used as an index, providing external anonymization while preserving a link to the original plan in the treatment planning system if needed subsequently. A series of Python scripts were used to extract DVH data for display or cohort median values from the MySQL database. Statistical comparisons between patient cohorts were performed using a web interface built with JavaScript allowing interactive and dynamic visualization. In our case, Cohort 1 corresponded to the preintervention dataset, which provided the constraints, and cohort 2 corresponded to the postintervention dataset.

The DVHR consists of three key elements: a MySQL database to store the DVH data, a web‐based user interface, and a series of Python scripts to import, load, and analyze the data. Figure 1 depicts the data flow through the DVHR as used in the presently described project. Patient information is filtered, anonymized, and imported into the database using a custom Python script. Data incorporated from each plan into the DVHR include the prescription dose and fractionation scheme, as well as organ volume (in cm^3^), absolute and relative DVH points, and the mean, median, maximum, and minimum doses for all targets and OAR structures.

Data within the DVHR may be grouped into “cohorts” and summary statistics may be used to compare and contrast these cohorts.

### 2.A Intervention

Beginning in 2014, our institution used the DVHR to establish CSI planning constraints based on our previous planning experience. Craniospinal irradiation plans were imported from the treatment planning system [Eclipse by Varian Medical Systems (Palo Alto, CA)] into the DVHR if the prescription dose was 36 Gy in 20 fractions. The plans of nine patients treated for CSI between 2009 and 2014 met these criteria and were accessible for the analysis. Using the dosimetric information of these patients, the population mean and median dose (along with the standard deviation) were calculated for 10 OARs: bilateral lungs, both kidneys, liver, heart, stomach, esophagus, trachea, and thyroid gland. New planning constraints for plan evaluation were established based on the median values and were used by the planners as the initial optimization criteria in plan optimization.

From 2014 onwards, physicians and dosimetrists planned new CSI patients prescribed 36 Gy in 20 fractions using the DVHR‐derived constraints. By early 2020, nine new patients had been treated and their data incorporated into the DVHR, at which point we evaluated the impact of the DVHR‐derived constraints on clinical practice.

### 2.B Evaluation

We undertook a pre–post analysis to determine the usefulness of the DVHR as a tool to monitor CSI treatment planning at our center. Two cohorts of CSI plans (using the standard prescription of 36 Gy in 20 fractions) were examined: ten plans from before the DVHR‐derived constraints were implemented in the clinic (preconstraints, incorporating the nine plans used to derive the constraints and one additional plan) and nine plans from after the implementation (postconstraints).

**FIG. 2:**
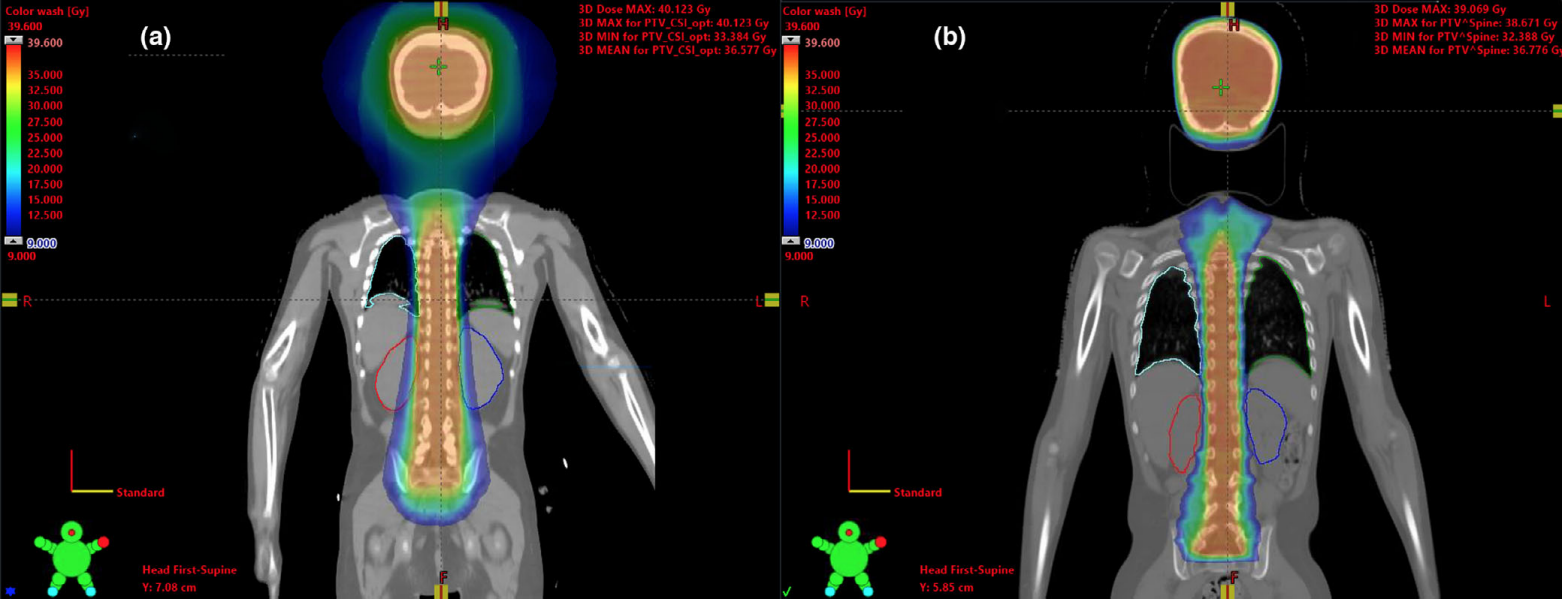
(a) Isodose comparison of a preconstraints patient treated with TomoTherapy and (b) a postconstraints patient treated with the IMRT technique. It is clear that the dose to the kidneys and lungs is reduced postintervention.

Plans included in this study were treated with two different radiation therapy modalities: Helical TomoTherapy (TomoTherapy Inc., Madison, WI) before May 2015 and intensity‐modulated radiation therapy (IMRT) using a linear accelerator (TrueBeam by Varian Medical Systems, Palo Alto, CA)^21^, ^22^ after May 2015. All patients in the preconstraints cohort and two in the postconstraints cohort were treated with TomoTherapy using 6 MV beam energy, 5 cm collimator size, and 0.3 pitch, while the remaining seven patients in the postconstraints cohort were treated with static gantry IMRT using the derived DVHR constraints for guidance, 6 MV beam, and 3 isocenters: superior, middle, and inferior. The reason for the change in technique was the move of our radiation therapy department to a new purpose‐built comprehensive cancer center in May 2015 where Helical TomoTherapy was not available. For illustration purposes, Fig. 2 displays the isodose comparison of two different patients treated with (a) TomoTherapy and (b) IMRT.

### 2.C Dose reduction examination

With the aim of visually observing pre–post changes for specific dosimetric points, the mean DVH and its standard uncertainty was plotted for each OAR‐cohort combination. This allowed us to quickly and qualitatively determine across the board if there was any reduction in the dose delivered to the OARs due to the intervention of the DVHR‐derived constraints.

In order to determine if use of the DVHR‐derived constraints led to a change in practice, the central tendencies (mean and median) and spread (standard deviation and interquartile range) of the D_mean_ values of each OAR were compared before and after the intervention, as any reductions postintervention could indicate an improvement in practice. D_mean_ was selected for this exercise as it provides a good summary of the dose to the whole structure and is widely applicable to OARs. Violin plots were used to evaluate changes in the median and interquartile ranges of the D_mean_ value of the population for each OAR. This method of plotting numeric data is similar to box plots with the advantage of also showing the probability density of the data at each dose value.^23^, ^24^

Due to the change in treatment modality, it was important to investigate if any changes in OAR sparing and treatment planning standardization after 2014 were due to the modality change or due to the use of the DVHR‐derived constraints. For this reason, we used timeline plots to examine the change in OAR dose over time in order to determine if any observed improvements began before the switch to IMRT.

### 2.D Mann–Whitney *U* tests

Mann–Whitney *U* tests were used to assess the pre‐ and post‐DVHR cohorts at each of the specific dosimetric points to determine if the observed changes in summary statistics were statistically significant.^25^, ^26^ We selected this nonparametric test of the null hypothesis (i.e., that there is no difference between cohorts) over others as our data are not normally distributed and our sample size is small.^27^ Eight tests, corresponding to V_5Gy_, V_10Gy_, V_15Gy_, V_20Gy_, D_mean_, D_median_, D_min_, and D_max_, comparing the pre‐ and postconstraints cohorts, were performed.

### 2.E Means and confidence intervals

In general, sample size plays an impactful role in the results obtained from hypothesis testing evaluations. Therefore, Mann–Whitney *U* tests, although insightful, may not be sufficient to adequately demonstrate the significance of changes in practice.^28^, ^29^, ^30^ Because of this, we also compared the pre‐ and post‐DVHR cohorts with respect to their population mean scores for all dosimetric points (V_5Gy_, V_10Gy_, V_15Gy_, V_20Gy_, D_mean_, D_median_, D_min_, and D_max_) and calculated the confidence intervals of the differences between them. The evaluation of the difference in means between cohorts at the 95% confidence interval was computed using the appropriate t distribution for the selected confidence level and the standard uncertainty of the point estimate.^31^

The execution of all statistical analyses was conducted using a custom‐written Python script incorporating a two‐tailed (*P* < 0.05) significance level.

## 3 RESULTS

### 3.A Intervention

The DVH constraints derived in 2014 are shown in Table 1 for all ten OARs (the lungs, kidneys, liver, heart, stomach, esophagus, trachea, and thyroid gland) examined in this study.

**TABLE 1:** OAR constraint values for CSI prescriptions of 36 Gy in 20 fractions, as derived from the median DVH values of the nine previously treated plans entered into the DVHR in 2014. OARs such as cochleas, eyes, pituitary, and chiasm that display 100% volume at V_5Gy_ and V_10Gy_, were not included within the DVHR analysis.

Organs at risk	Relative volume [%]		
	V_5Gy_	V_10Gy_	V_20Gy_
Heart	92	23	0
Left lung	52	11	1
Right lung	68	17	4
Left kidney	62	15	0
Right kidney	75	13	0
Esophagus	100	100	35
Liver	63	21	0
Stomach	100	26	0
Trachea	100	100	22
Thyroid	100	91	2

These constraints were added to the ARIA Oncology Information System (Varian Medical Systems, Palo Alto, CA) as a template easily accessible on a so‐called "CT Planning Sheet'' that listed instructions from physicians to dosimetrists for planning new patients from 2014 onwards. Prior to the use of these constraints, physicians’ instructions were simply to keep OAR doses as low as reasonably achievable.

### 3.B Evaluation

**FIG. 3:**
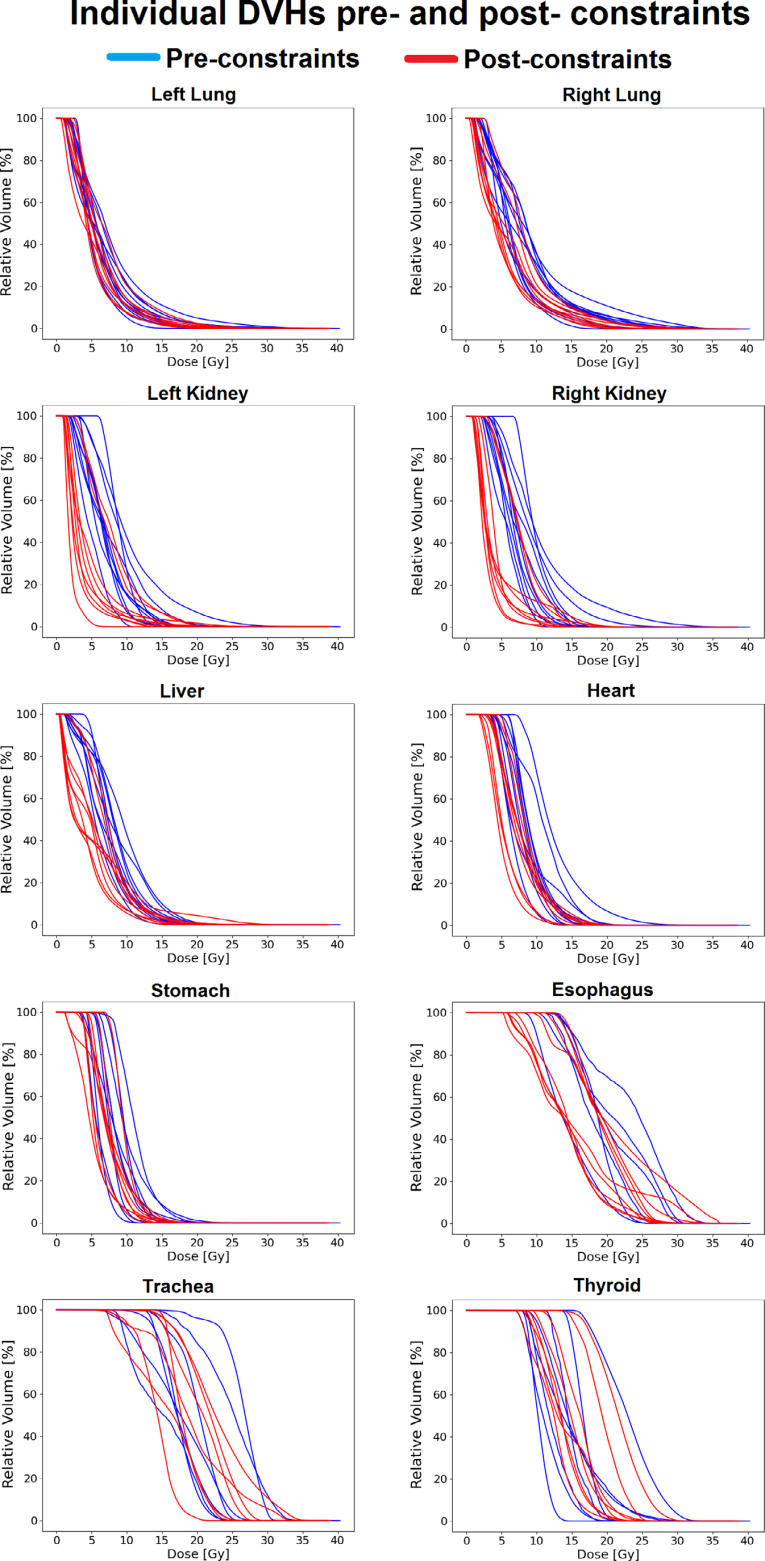
Individual DVHs before and after the intervention of the DVHR for all OARs examined. Each line represents a different anonymized patient treated with CSI at our center.

All individual DVHs for each patient OAR, before and after implementation of the planning constraints, are shown in Fig. 3. Large structures such as the heart, lungs, kidneys, liver, and stomach displayed a clear reduction in dose in the postconstraints period compared to the preconstraints period. Other structures, particularly smaller structures close to the target volume, had less or no reductions in dose after implementation of the DVHR.

### 3.C Dose reduction examination

**FIG. 4:**
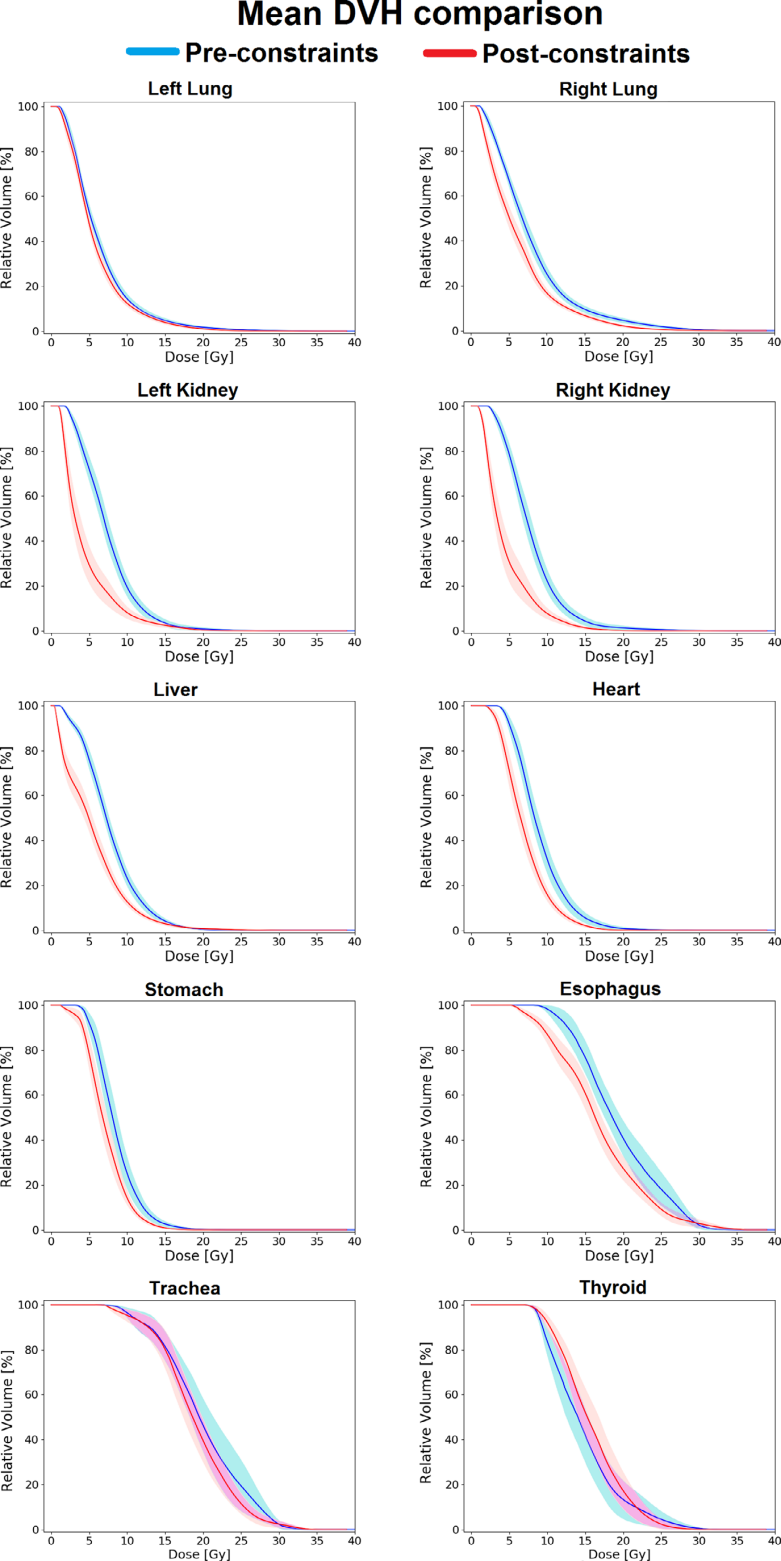
Comparison of population‐mean DVHs of all OARs before and after the intervention of the DVHR. Shaded regions indicate the standard uncertainty of the mean.

The population‐mean DVHs of the pre‐ and postconstraints cohorts are shown in Fig. 4. The heart, right lung, liver, and kidneys had the most dramatic decrease in their mean DVHs postconstraints, whereas the left lung, esophagus, stomach, and trachea demonstrated less obvious or minimal reductions. The thyroid gland was the only OAR that displayed an increased dose after the intervention, unlike all other organs which displayed either a lower or similar population‐mean DVH in the postconstraints cohort.

**FIG. 5:**
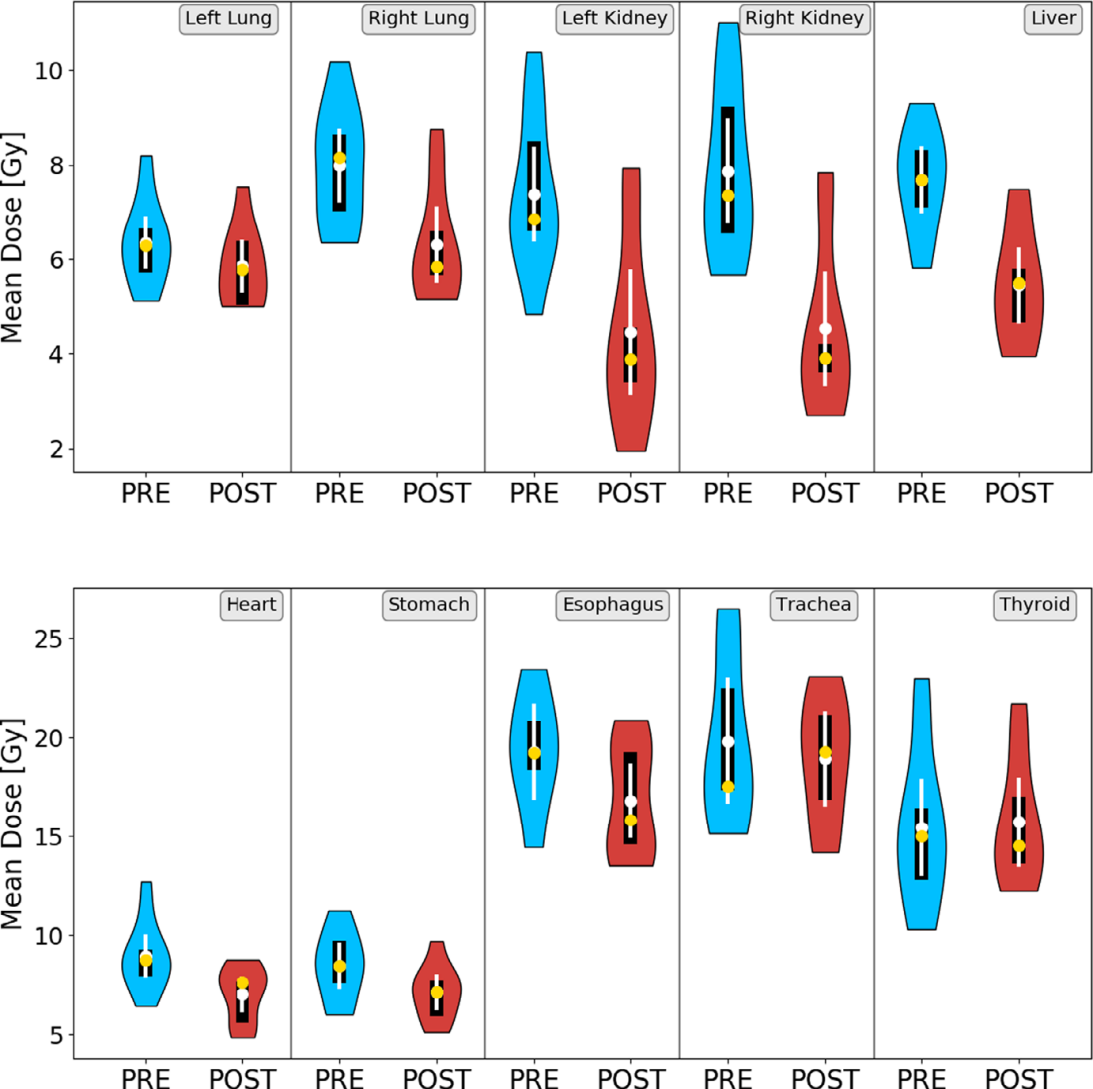
Violin plots of the D_mean_ delivered to all OARs before and after the intervention of the DVHR. Mean and median are demarcated by white and yellow dots, respectively. Additionally, the 95% confidence intervals are shown as white lines and the interquartile ranges as black lines.

Following the implementation of the constraints in 2014, the interquartile ranges of seven of ten OARs decreased for the D_mean_ parameter: the right lung, kidneys, liver, stomach, thyroid, and trachea (Fig. 5). The right kidney and trachea had the most pronounced changes in their interquartile ranges, decreasing from 2.7 to 0.61 Gy and from 5.2 to 4.2 Gy, respectively. In contrast, the esophagus, heart, and left lung increased their interquartile ranges postconstraints by 2.2, 0.7, and 0.4 Gy, respectively. This was also consistent with the spreads seen visually in the individual DVHs (Fig. 3).

Except for the trachea and the thyroid gland, all OARs had a reduced population mean and median of the D_mean_ value postintervention (Fig. 5). The most dramatic decrease in population median D_mean_ was for the liver, left and right kidneys, which reduced by 29%, 43%, and 47%, respectively.

Supplementary material presented at the end of this manuscript contains detailed information regarding the population median of D_mean_ and the interquartile ranges for all OARs.

**FIG. 6:**
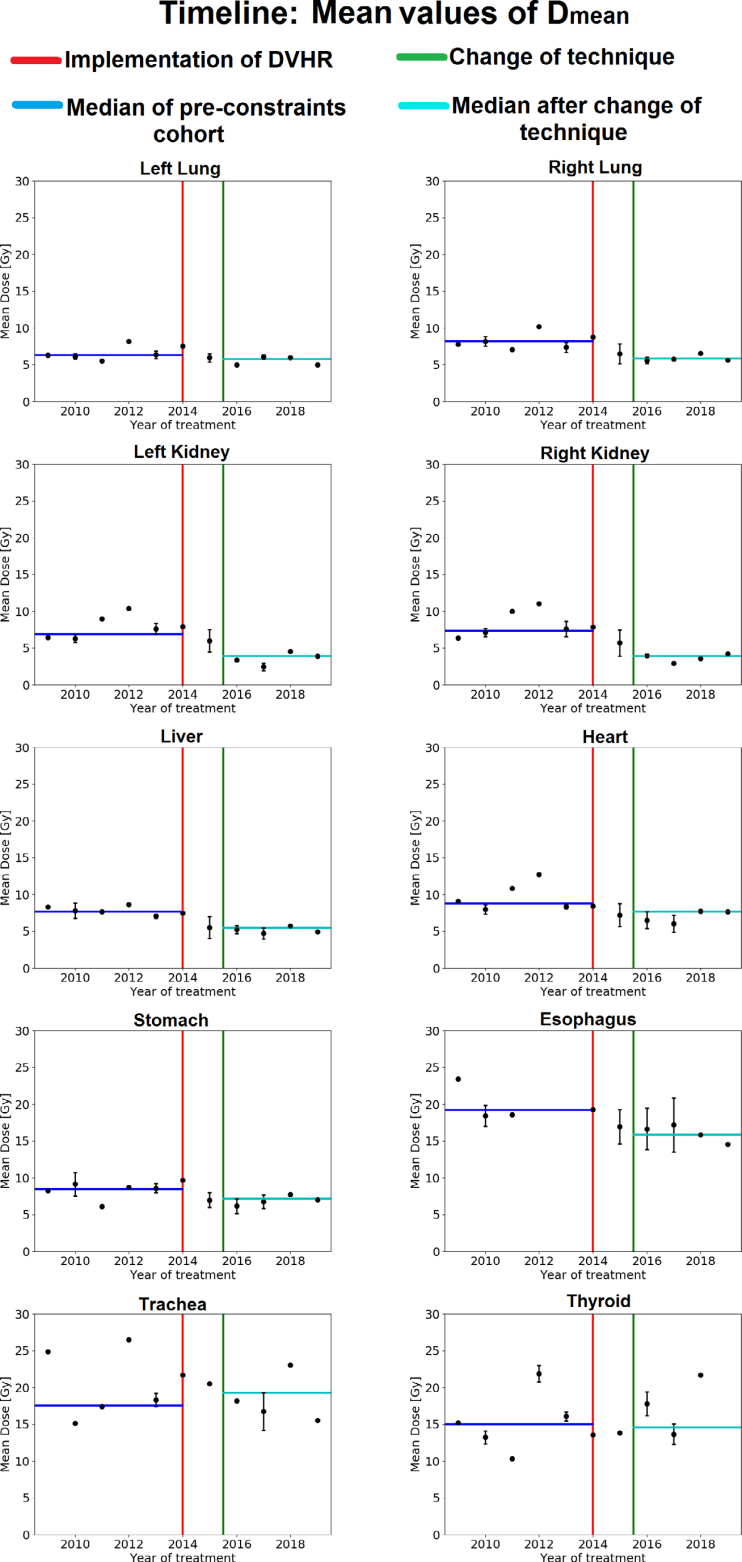
Time scale evaluation of the population mean values of D_mean_ over the years of the study. A total of 19 patients were used in each figure, separated into 12 yr‐groups, from 2009 to 2019. Each black dot represents the mean value of the D_mean_ values in each year‐group. The error bars represent the standard deviation of the mean. For year‐groups with only one data point, the standard deviation of the mean was not computed. The vertical red line represents the year in which the DVHR was implemented in the clinic and the green line refers to the beginning of CSI treatments using the IMRT technique. Horizontal lines represent the median value of all D_mean_ points before the intervention (Blue) and after the change of technique (Cyan).

The mean values of D_mean_ for each year of the study are depicted in Fig. 6 as temporal trends for all OARs. The mean value of D_mean_ reduced after the constraints were introduced but before the change in technique for all structures except for the thyroid gland.

### 3.D Mann–Whitney *U* tests

**FIG. 7:**
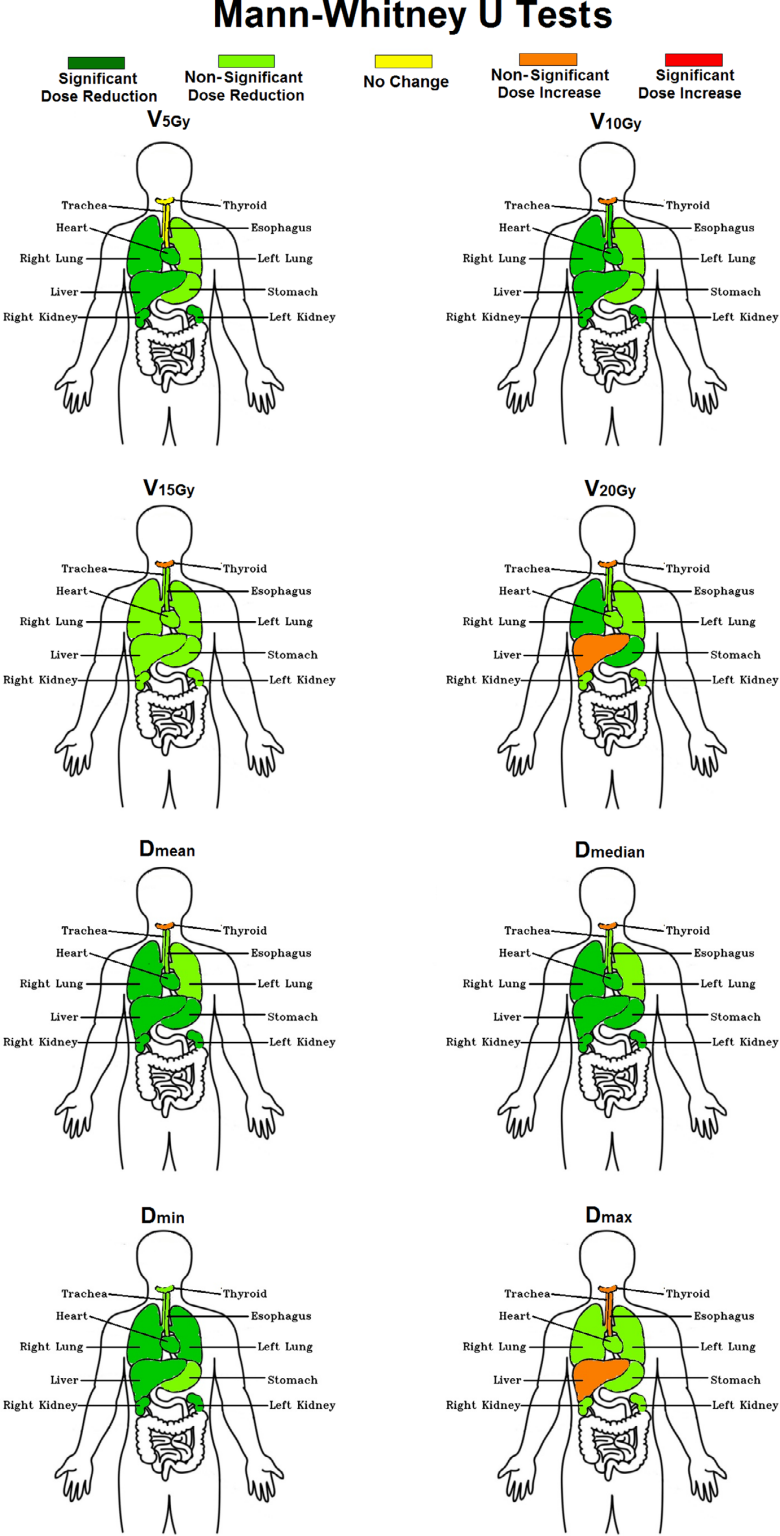
Hypothesis test results after performing the two‐tailed (*P* < 0.05 deemed statistically significant) Mann–Whitney *U* test on the ten OARs examined. The parameters evaluated were the V_5Gy_, V_10Gy_, V_15Gy_, V_20Gy_, D_mean_, D_median_, D_min_, and D_max_ values delivered to each structure. p‐values and dose reduction results were combined to display five possible colors for each OAR: Green (statistically significant dose reduction), light green (statistically nonsignificant dose reduction), yellow (statistically no change), orange (statistically nonsignificant dose increase), and red (statistically significant dose increase).

According to the Mann–Whitney *U* tests (*results shown in* Fig. 7), the introduction of the new dosimetric constraints led to statistically significant reductions in dose to the heart, right lung, both kidneys, and liver. This can be observed from the significant decreases in V_5Gy_, V_10Gy_, D_mean_, D_median_, and D_min_ for these structures. Although there were some reductions in V_5Gy_, V_10Gy_, V_15Gy_, V_20Gy_ for the left lung, esophagus, and trachea postconstraints, these reductions were not statistically significant. Overall, dose to OARs remained similar or was reduced nonsignificantly for most structures. However, V_20Gy_ to the liver, D_max_ to the esophagus and trachea, and V_10Gy_, V_15Gy_, V_20Gy_, D_mean_, D_median_, and D_max_ to the thyroid gland, all increased after constraints were implemented, although those increases were not statistically significant.

### 3.E Means and confidence intervals

Ninety‐five percent confidence intervals for the difference of means between the pre‐ and postconstraints cohorts were calculated to validate the conclusions of the Mann–Whitney *U* tests. Results were 94% consistent, with 75 of the 80 evaluations agreeing between the two tests. The disagreements pertained to the esophageal V_10Gy_, left lung D_min_, and the stomach V_20Gy_, D_mean_, and D_median_. In all cases of disagreement, the 95% confidence interval results suggested there were no significant differences between the pre‐ and postconstraints cohorts, whereas the Mann–Whitney U tests indicated that there were. Full details of this analysis are presented in the supplementary material.

## 4 DISCUSSION

Standardization of radiation therapy planning and delivery is an important way to reduce the risk of long‐term side effects in cancer patients. In this work, we examined whether or not an intervention in clinical practice of using dosimetric constraints derived from retrospective data can provide continuous monitoring of pediatric CSI treatment planning and potentially lead to standardization.

Overall, we found that the implementation of constraints derived using our DVHR was associated with a significant reduction in the dose delivered to the heart, right lung, stomach, liver, and bilateral kidneys across all dosimetric parameters examined. Nonsignificant dose reductions occurred in all other organs, except for the trachea, esophagus, and thyroid gland, which displayed increased doses for various parameters postconstraints, albeit not significantly so. One possible explanation for why certain OARs experienced greater dose reductions than others is organ location and size. The heart, lungs, and kidneys are composed of volume that resides further from the target spinal column whereas the trachea, esophagus, and thyroid gland lie in close proximity to it. Previous studies have examined the OAR sparing that TomoTherapy and IMRT can achieve for CSI patients.^3^, ^7^, ^22^, ^32^ While both techniques can achieve good OAR sparing, IMRT has been demonstrated to better reduce dose to OARs distant from the target volume, whereas Tomotherapy was shown to better spare OARs in close target proximity^7^, ^9^, ^22^ Given our change in technique postconstraints, this could have contributed to dose to the trachea and thyroid gland increasing after the constraints were introduced and may also have influenced the sparing of other organs like the kidneys.

We found that the majority of OARs (right lung, liver, stomach, thyroid, and kidneys) presented a reduction of D_mean_ in the measure of population central tendency (median) and the spread (interquartile range) postintervention. Before the use of the DVHR‐derived constraints, physicians’ instructions at our institution were typically to keep OAR doses as low as reasonably achievable, which allowed a wide range of dose possibilities depending on the expertise and experience of the planner. Having pre‐established constraints provided a clear and standardized way to convey planning instructions to planners.

We recognize that the small sample size of our patient cohort limits the strength of the conclusions we can draw from our results, which is an inherent limitation of single‐institution medulloblastoma research. Only 0.2% of all cancer patients are diagnosed with a central nervous system tumor,^33^ meaning only a handful can be expected to be treated at a single institution every year. Multi‐institutional research collaborations such as PENTEC,^11^ will not only allow more precise statistical analyses but also will allow consideration of other potentially influencing factors such as patient age and sex.

The change of treatment technique at our institution in 2015 may have had a confounding effect on the results of this study. However, some improvements in practice standardization were observable prior to the change for all OARs, except the thyroid gland, using timeline trends that show the trend of mean dose over the years. Also, the reduction in dose variability is more likely attributable to consistent planning than to a change in technique.

We can also attest that the use of consistent DVHR‐derived constraints helped facilitate the introduction of the IMRT technique in 2015. It provided a set of existing dosimetric expectations for the new technique and thus provided useful guidance to our planners as they developed and implemented it into clinical practice. In itself, this demonstrates an important benefit of the use of standardized treatment planning constraints in clinical change management.

The results of our study indicate that the clinical use of a DVHR can enable continuous quality monitoring and facilitate improvement in clinical practice. Using a DVHR can help radiation therapy centers with smaller pediatric practices to approach best‐achievable monitoring in treatment planning while extensive multi‐institutional research initiatives, such as PENTEC,^11^ draw up more generally applicable guidelines.

As future work, quantitative treatment outcomes will be extracted from the medical records and used to assess the clinical impact of the reduced dose to the OARs. Use of the DVHR will also be applied to other radiotherapy techniques and treatment sites that lack monitoring of practice.

## 5 CONCLUSION

In this research, we demonstrated how monitoring of CSI planning may be achieved using dosimetric planning constraints derived from previous treatment plans. We provided DVHR‐derived constraints to physicians and dosimetrists to plan and evaluate new CSI patients. The DVHR allowed us to compare planning practice before and after the intervention and to determine if practice had improved. Although we cannot conclude the DVHR‐derived constraints were entirely responsible for all improvements, due to the confounding factor of a change in treatment technique, guidance for the change in technique was itself provided by the DVHR‐derived constraints, which certainly contributed to monitoring of practice across techniques.

## CONFLICT OF INTEREST

There is no conflict of interest to disclose.

## Supporting information


**Data S1**. The following tables provide detailed information regarding the interquartile ranges, the population median of D_mean_, the Mann‐Whitney U tests, and the mean differences at 95% confidence intervals for the statistical evaluation between the pre‐ and post‐cohorts in this study for all OARs.
