# Supplementary material for: Implementation of a DVH Registry to provide constraints and continuous quality monitoring for pediatric CSI treatment planning
Source: J Appl Clin Med Phys. 2020 Dec 14;22(1):191–202. doi: 10.1002/acm2.13131 (PMC7856485; doi:10.1002/acm2.13131)
Supplement: Supplementary file 1 — Data S1. The following tables provide detailed information regarding the interquartile ranges, the population median of Dmean, the Mann‐Whitney U tests, and the mean differences at 95% confidence intervals for the statistical evaluation between the pre‐ and post‐cohorts in this study for all OARs. [file ACM2-22-191-s001.docx]

**Supplementary material to the manuscript: “Standardization of CSI Treatment Planning and its Evaluation Using a DVH Registry” by Esteban Sepulveda, MSc, Haley Patrick, MSc, Carolyn Freeman, MD, and John Kildea, Ph.D. Article submitted to the Journal of Applied Clinical Medical Physics.**

The following tables provide detailed information regarding the interquartile ranges, the population median of D_mean_, the Mann-Whitney U tests, and the mean differences at 95% confidence intervals for the statistical evaluation between the pre- and post-cohorts in this study for all OARs.

Table 1 - Interquartile ranges in Gy for all OARs regarding the mean dose delivered to the structure.

|  | **Dmean: Interquartile Ranges in Gy** | | | |
| --- | --- | --- | --- | --- |
|  | **PRE** | **POST** | **PRE-POST** | **Reduced by:** |
| **Heart** | 1.35 | 2.09 | -0.75 | -55% |
| **Left Lung** | 0.94 | 1.35 | -0.42 | -45% |
| **Right Lung** | 1.64 | 0.94 | 0.70 | 43% |
| **Left Kidney** | 1.91 | 1.15 | 0.76 | 40% |
| **Right Kidney** | 2.68 | 0.62 | 2.07 | 77% |
| **Liver** | 1.22 | 1.14 | 0.08 | 7% |
| **Stomach** | 2.15 | 1.81 | 0.34 | 16% |
| **Esophagus** | 2.47 | 4.73 | -2.25 | -91% |
| **Trachea** | 5.18 | 4.25 | 0.92 | 18% |
| **Thyroid** | 3.62 | 3.37 | 0.26 | 7% |

Table 2 - Population median of D_mean_ for all OARs in Gy unit.

|  | **Dmean: Median values in Gy** | | | |
| --- | --- | --- | --- | --- |
|  | **PRE** | **POST** | **PRE-POST** | **Reduced by:** |
| **Heart** | 8.74 | 7.63 | 1.11 | 13% |
| **Left Lung** | 6.28 | 5.79 | 0.49 | 8% |
| **Right Lung** | 8.15 | 5.84 | 2.31 | 28% |
| **Left Kidney** | 6.86 | 3.89 | 2.97 | 43% |
| **Right Kidney** | 7.36 | 3.90 | 3.46 | 47% |
| **Liver** | 7.69 | 5.49 | 2.21 | 29% |
| **Stomach** | 8.47 | 7.15 | 1.32 | 16% |
| **Esophagus** | 19.21 | 15.84 | 3.37 | 18% |
| **Trachea** | 17.54 | 19.26 | -1.72 | -10% |
| **Thyroid** | 15.00 | 14.53 | 0.46 | 3% |

Table 3 - P-values and mean difference at 95% confidence interval for the left lung at all dosimetric points evaluated in this study.

| **Left Lung** | | | | | |
| --- | --- | --- | --- | --- | --- |
| **Dosimetric parameter** | **Evaluation** | **Result** | **Dosimetric parameter** | **Evaluation** | **Result** |
| **V _5Gy_** | **MWU test result** | NOT Sig diff. | **D_mean_** | **MWU test result** | NOT Sig diff. |
|  | **P-Value** | 0.103 |  | **P-Value** | 0.103 |
|  | **Mean Diff.** | 5.14 |  | **Mean Diff.** | 0.5 |
|  | **Conf. Inter. 95%** | [-3.63 to 13.91] |  | **Conf. Inter. 95%** | [-0.35 to 1.36] |
|  | **Population mean post-constraints** | Reduced |  | **Population mean post-constraints** | Reduced |
| **V _10Gy_** | **MWU test result** | NOT Sig diff. | **D_median_** | **MWU test result** | NOT Sig diff. |
|  | **P-Value** | 0.388 |  | **P-Value** | 0.077 |
|  | **Mean Diff.** | 2.02 |  | **Mean Diff.** | 0.52 |
|  | **Conf. Inter. 95%** | [-3.43 to 7.46] |  | **Conf. Inter. 95%** | [-0.23 to 1.27] |
|  | **Population mean post-constraints** | Reduced |  | **Population mean post-constraints** | Reduced |
| **V _15Gy_** | **MWU test result** | NOT Sig diff. | **D_min_** | **MWU test result** | Sig diff. |
|  | **P-Value** | 0.298 |  | **P-Value** | 0.028 |
|  | **Mean Diff.** | 0.84 |  | **Mean Diff.** | 0.35 |
|  | **Conf. Inter. 95%** | [-1.76 to 3.45] |  | **Conf. Inter. 95%** | [-0.10 to 0.80] |
|  | **Population mean post-constraints** | Reduced |  | **Population mean post-constraints** | Reduced |
| **V _20Gy_** | **MWU test result** | NOT Sig diff. | **D_max_** | **MWU test result** | NOT Sig diff. |
|  | **P-Value** | 0.118 |  | **P-Value** | 0.065 |
|  | **Mean Diff.** | 0.69 |  | **Mean Diff.** | 2.45 |
|  | **Conf. Inter. 95%** | [-0.46 to 1.84] |  | **Conf. Inter. 95%** | [-2.25 to 7.15] |
|  | **Population mean post-constraints** | Reduced |  | **Population mean post-constraints** | Reduced |

Table 4 - P-values and mean difference at 95% confidence interval for the right lung at all dosimetric points evaluated in this study.

| **Right Lung** | | | | | |
| --- | --- | --- | --- | --- | --- |
| **Dosimetric parameter** | **Evaluation** | **Result** | **Dosimetric parameter** | **Evaluation** | **Result** |
| **V _5Gy_** | **MWU test result** | Sig diff. | **D_mean_** | **MWU test result** | Sig diff. |
|  | **P-Value** | 0.015 |  | **P-Value** | 0.008 |
|  | **Mean Diff.** | 16.47 |  | **Mean Diff.** | 1.68 |
|  | **Conf. Inter. 95%** | [5.31 to 27.63] |  | **Conf. Inter. 95%** | [0.46 to 2.89] |
|  | **Population mean post-constraints** | Reduced |  | **Population mean post-constraints** | Reduced |
| **V _10Gy_** | **MWU test result** | Sig diff. | **D_median_** | **MWU test result** | Sig diff. |
|  | **P-Value** | 0.028 |  | **P-Value** | 0.005 |
|  | **Mean Diff.** | 8.53 |  | **Mean Diff.** | 1.77 |
|  | **Conf. Inter. 95%** | [0.75 to 16.30] |  | **Conf. Inter. 95%** | [0.47 to 3.07] |
|  | **Population mean post-constraints** | Reduced |  | **Population mean post-constraints** | Reduced |
| **V _15Gy_** | **MWU test result** | NOT Sig diff. | **D_min_** | **MWU test result** | Sig diff. |
|  | **P-Value** | 0.056 |  | **P-Value** | 0.004 |
|  | **Mean Diff.** | 2.78 |  | **Mean Diff.** | 0.6 |
|  | **Conf. Inter. 95%** | [-0.91 to 6.46] |  | **Conf. Inter. 95%** | [0.12 to 1.08] |
|  | **Population mean post-constraints** | Reduced |  | **Population mean post-constraints** | Reduced |
| **V _20Gy_** | **MWU test result** | Sig diff. | **D_max_** | **MWU test result** | NOT Sig diff. |
|  | **P-Value** | 0.015 |  | **P-Value** | 0.174 |
|  | **Mean Diff.** | 2.46 |  | **Mean Diff.** | 2.38 |
|  | **Conf. Inter. 95%** | [0.01 to 4.92] |  | **Conf. Inter. 95%** | [-2.42 to 7.18] |
|  | **Population mean post-constraints** | Reduced |  | **Population mean post-constraints** | Reduced |

Table 5 - P-values and mean difference at 95% confidence interval for the left kidney at all dosimetric points evaluated in this study.

| **Left Kidney** | | | | | |
| --- | --- | --- | --- | --- | --- |
| **Dosimetric parameter** | **Evaluation** | **Result** | **Dosimetric parameter** | **Evaluation** | **Result** |
| **V _5Gy_** | **MWU test result** | Sig diff. | **D_mean_** | **MWU test result** | Sig diff. |
|  | **P-Value** | 0.003 |  | **P-Value** | 0.006 |
|  | **Mean Diff.** | 42.63 |  | **Mean Diff.** | 2.93 |
|  | **Conf. Inter. 95%** | [22.14 to 63.12] |  | **Conf. Inter. 95%** | [1.17 to 4.69] |
|  | **Population mean post-constraints** | Reduced |  | **Population mean post-constraints** | Reduced |
| **V _10Gy_** | **MWU test result** | Sig diff. | **D_median_** | **MWU test result** | Sig diff. |
|  | **P-Value** | 0.019 |  | **P-Value** | 0.006 |
|  | **Mean Diff.** | 11.36 |  | **Mean Diff.** | 3.22 |
|  | **Conf. Inter. 95%** | [0.78 to 21.93] |  | **Conf. Inter. 95%** | [1.49 to 4.95] |
|  | **Population mean post-constraints** | Reduced |  | **Population mean post-constraints** | Reduced |
| **V _15Gy_** | **MWU test result** | NOT Sig diff. | **D_min_** | **MWU test result** | Sig diff. |
|  | **P-Value** | 0.451 |  | **P-Value** | 0.004 |
|  | **Mean Diff.** | 1.02 |  | **Mean Diff.** | 1.17 |
|  | **Conf. Inter. 95%** | [-3.09 to 5.14] |  | **Conf. Inter. 95%** | [0.25 to 2.10] |
|  | **Population mean post-constraints** | Reduced |  | **Population mean post-constraints** | Reduced |
| **V _20Gy_** | **MWU test result** | NOT Sig diff. | **D_max_** | **MWU test result** | NOT Sig diff. |
|  | **P-Value** | 0.194 |  | **P-Value** | 0.388 |
|  | **Mean Diff.** | 0.5 |  | **Mean Diff.** | 0.91 |
|  | **Conf. Inter. 95%** | [-1.04 to 2.04] |  | **Conf. Inter. 95%** | [-5.06 to 6.89] |
|  | **Population mean post-constraints** | Reduced |  | **Population mean post-constraints** | Reduced |

Table 6 - P-values and mean difference at 95% confidence interval for the right kidney at all dosimetric points evaluated in this study.

| **Right Kidney** | | | | | |
| --- | --- | --- | --- | --- | --- |
| **Dosimetric parameter** | **Evaluation** | **Result** | **Dosimetric parameter** | **Evaluation** | **Result** |
| **V _5Gy_** | **MWU test result** | Sig diff. | **D_mean_** | **MWU test result** | Sig diff. |
|  | **P-Value** | 0.001 |  | **P-Value** | 0.004 |
|  | **Mean Diff.** | 47.85 |  | **Mean Diff.** | 3.33 |
|  | **Conf. Inter. 95%** | [27.25 to 68.44] |  | **Conf. Inter. 95%** | [1.57 to 5.10] |
|  | **Population mean post-constraints** | Reduced |  | **Population mean post-constraints** | Reduced |
| **V _10Gy_** | **MWU test result** | Sig diff. | **D_median_** | **MWU test result** | Sig diff. |
|  | **P-Value** | 0.010 |  | **P-Value** | 0.004 |
|  | **Mean Diff.** | 14.42 |  | **Mean Diff.** | 3.5 |
|  | **Conf. Inter. 95%** | [2.54 to 26.30] |  | **Conf. Inter. 95%** | [1.82 to 5.19] |
|  | **Population mean post-constraints** | Reduced |  | **Population mean post-constraints** | Reduced |
| **V _15Gy_** | **MWU test result** | NOT Sig diff. | **D_min_** | **MWU test result** | Sig diff. |
|  | **P-Value** | 0.135 |  | **P-Value** | 0.001 |
|  | **Mean Diff.** | 2.99 |  | **Mean Diff.** | 1.59 |
|  | **Conf. Inter. 95%** | [-1.54 to 7.52] |  | **Conf. Inter. 95%** | [0.53 to 2.65] |
|  | **Population mean post-constraints** | Reduced |  | **Population mean post-constraints** | Reduced |
| **V _20Gy_** | **MWU test result** | NOT Sig diff. | **D_max_** | **MWU test result** | NOT Sig diff. |
|  | **P-Value** | 0.480 |  | **P-Value** | 0.196 |
|  | **Mean Diff.** | 1.16 |  | **Mean Diff.** | 2.59 |
|  | **Conf. Inter. 95%** | [-0.98 to 3.29] |  | **Conf. Inter. 95%** | [-3.11 to 8.29] |
|  | **Population mean post-constraints** | Reduced |  | **Population mean post-constraints** | Reduced |

Table 7 - P-values and mean difference at 95% confidence interval for the liver at all dosimetric points evaluated in this study.

| **Liver** | | | | | |
| --- | --- | --- | --- | --- | --- |
| **Dosimetric parameter** | **Evaluation** | **Result** | **Dosimetric parameter** | **Evaluation** | **Result** |
| **V _5Gy_** | **MWU test result** | Sig diff. | **D_mean_** | **MWU test result** | Sig diff. |
|  | **P-Value** | 0.002 |  | **P-Value** | 0.001 |
|  | **Mean Diff.** | 26.58 |  | **Mean Diff.** | 2.24 |
|  | **Conf. Inter. 95%** | [11.91 to 41.25] |  | **Conf. Inter. 95%** | [1.08 to 3.40] |
|  | **Population mean post-constraints** | Reduced |  | **Population mean post-constraints** | Reduced |
| **V _10Gy_** | **MWU test result** | Sig diff. | **D_median_** | **MWU test result** | Sig diff. |
|  | **P-Value** | 0.014 |  | **P-Value** | 0.002 |
|  | **Mean Diff.** | 10.48 |  | **Mean Diff.** | 2.73 |
|  | **Conf. Inter. 95%** | [2.15 to 18.82] |  | **Conf. Inter. 95%** | [1.23 to 4.23] |
|  | **Population mean post-constraints** | Reduced |  | **Population mean post-constraints** | Reduced |
| **V _15Gy_** | **MWU test result** | NOT Sig diff. | **D_min_** | **MWU test result** | Sig diff. |
|  | **P-Value** | 0.240 |  | **P-Value** | 0.005 |
|  | **Mean Diff.** | 1.19 |  | **Mean Diff.** | 0.85 |
|  | **Conf. Inter. 95%** | [-1.52 to 3.90] |  | **Conf. Inter. 95%** | [0.23 to 1.46] |
|  | **Population mean post-constraints** | Reduced |  | **Population mean post-constraints** | Reduced |
| **V _20Gy_** | **MWU test result** | NOT Sig diff. | **D_max_** | **MWU test result** | NOT Sig diff. |
|  | **P-Value** | 0.430 |  | **P-Value** | 0.362 |
|  | **Mean Diff.** | -0.37 |  | **Mean Diff.** | -1.28 |
|  | **Conf. Inter. 95%** | [-1.39 to 0.66] |  | **Conf. Inter. 95%** | [-6.97 to 4.41] |
|  | **Population mean post-constraints** | Increased |  | **Population mean post-constraints** | Increased |

Table 8 - P-values and mean difference at 95% confidence interval for the heart at all dosimetric points evaluated in this study.

| **Heart** | | | | | |
| --- | --- | --- | --- | --- | --- |
| **Dosimetric parameter** | **Evaluation** | **Result** | **Dosimetric parameter** | **Evaluation** | **Result** |
| **V _5Gy_** | **MWU test result** | Sig diff. | **D_mean_** | **MWU test result** | Sig diff. |
|  | **P-Value** | 0.012 |  | **P-Value** | 0.010 |
|  | **Mean Diff.** | 20.78 |  | **Mean Diff.** | 1.92 |
|  | **Conf. Inter. 95%** | [4.81 to 36.75] |  | **Conf. Inter. 95%** | [0.37 to 3.47] |
|  | **Population mean post-constraints** | Reduced |  | **Population mean post-constraints** | Reduced |
| **V _10Gy_** | **MWU test result** | Sig diff. | **D_median_** | **MWU test result** | Sig diff. |
|  | **P-Value** | 0.019 |  | **P-Value** | 0.015 |
|  | **Mean Diff.** | 15.7 |  | **Mean Diff.** | 1.9 |
|  | **Conf. Inter. 95%** | [0.38 to 31.02] |  | **Conf. Inter. 95%** | [0.35 to 3.45] |
|  | **Population mean post-constraints** | Reduced |  | **Population mean post-constraints** | Reduced |
| **V _15Gy_** | **MWU test result** | NOT Sig diff. | **D_min_** | **MWU test result** | Sig diff. |
|  | **P-Value** | 0.270 |  | **P-Value** | 0.004 |
|  | **Mean Diff.** | 3.43 |  | **Mean Diff.** | 1.6 |
|  | **Conf. Inter. 95%** | [-1.85 to 8.71] |  | **Conf. Inter. 95%** | [0.58 to 2.62] |
|  | **Population mean post-constraints** | Reduced |  | **Population mean post-constraints** | Reduced |
| **V _20Gy_** | **MWU test result** | NOT Sig diff. | **D_max_** | **MWU test result** | NOT Sig diff. |
|  | **P-Value** | 0.323 |  | **P-Value** | 0.484 |
|  | **Mean Diff.** | 0.71 |  | **Mean Diff.** | 0.63 |
|  | **Conf. Inter. 95%** | [-0.77 to 2.20] |  | **Conf. Inter. 95%** | [-3.25 to 4.50] |
|  | **Population mean post-constraints** | Reduced |  | **Population mean post-constraints** | Reduced |

Table 9 - P-values and mean difference at 95% confidence interval for the stomach at all dosimetric points evaluated in this study.

| **Stomach** | | | | | |
| --- | --- | --- | --- | --- | --- |
| **Dosimetric parameter** | **Evaluation** | **Result** | **Dosimetric parameter** | **Evaluation** | **Result** |
| **V _5Gy_** | **MWU test result** | NOT Sig diff. | **D_mean_** | **MWU test result** | Sig diff. |
|  | **P-Value** | 0.056 |  | **P-Value** | 0.032 |
|  | **Mean Diff.** | 13.72 |  | **Mean Diff.** | 1.33 |
|  | **Conf. Inter. 95%** | [-3.38 to 30.81] |  | **Conf. Inter. 95%** | [-0.24 to 2.90] |
|  | **Population mean post-constraints** | Reduced |  | **Population mean post-constraints** | Reduced |
| **V _10Gy_** | **MWU test result** | NOT Sig diff. | **D_median_** | **MWU test result** | Sig diff. |
|  | **P-Value** | 0.166 |  | **P-Value** | 0.026 |
|  | **Mean Diff.** | 11.01 |  | **Mean Diff.** | 1.39 |
|  | **Conf. Inter. 95%** | [-5.31 to 27.34] |  | **Conf. Inter. 95%** | [-0.18 to 2.96] |
|  | **Population mean post-constraints** | Reduced |  | **Population mean post-constraints** | Reduced |
| **V _15Gy_** | **MWU test result** | NOT Sig diff. | **D_min_** | **MWU test result** | NOT Sig diff. |
|  | **P-Value** | 0.267 |  | **P-Value** | 0.067 |
|  | **Mean Diff.** | 1.75 |  | **Mean Diff.** | 1.1 |
|  | **Conf. Inter. 95%** | [-0.78 to 4.28] |  | **Conf. Inter. 95%** | [-0.35 to 2.54] |
|  | **Population mean post-constraints** | Reduced |  | **Population mean post-constraints** | Reduced |
| **V _20Gy_** | **MWU test result** | Sig diff. | **D_max_** | **MWU test result** | NOT Sig diff. |
|  | **P-Value** | 0.023 |  | **P-Value** | 0.268 |
|  | **Mean Diff.** | 0.19 |  | **Mean Diff.** | 0.53 |
|  | **Conf. Inter. 95%** | [-0.09 to 0.46] |  | **Conf. Inter. 95%** | [-3.30 to 4.36] |
|  | **Population mean post-constraints** | Reduced |  | **Population mean post-constraints** | Reduced |

Table 10 - P-values and mean difference at 95% confidence interval for the esophagus at all dosimetric points evaluated in this study.

| **Esophagus** | | | | | |
| --- | --- | --- | --- | --- | --- |
| **Dosimetric parameter** | **Evaluation** | **Result** | **Dosimetric parameter** | **Evaluation** | **Result** |
| **V _5Gy_** | **MWU test result** | No Change | **D_mean_** | **MWU test result** | NOT Sig diff. |
|  | **P-Value** | No p-value |  | **P-Value** | 0.108 |
|  | **Mean Diff.** | - |  | **Mean Diff.** | 2.49 |
|  | **Conf. Inter. 95%** | - |  | **Conf. Inter. 95%** | [-0.86 to 5.84] |
|  | **Population mean post-constraints** | Equal |  | **Population mean post-constraints** | Reduced |
| **V _10Gy_** | **MWU test result** | Sig diff. | **D_median_** | **MWU test result** | NOT Sig diff. |
|  | **P-Value** | 0.047 |  | **P-Value** | 0.188 |
|  | **Mean Diff.** | 11.25 |  | **Mean Diff.** | 2.9 |
|  | **Conf. Inter. 95%** | [-0.25 to 22.75] |  | **Conf. Inter. 95%** | [-0.60 to 6.40] |
|  | **Population mean post-constraints** | Reduced |  | **Population mean post-constraints** | Reduced |
| **V _15Gy_** | **MWU test result** | NOT Sig diff. | **D_min_** | **MWU test result** | NOT Sig diff. |
|  | **P-Value** | 0.158 |  | **P-Value** | 0.056 |
|  | **Mean Diff.** | 15.98 |  | **Mean Diff.** | 2.5 |
|  | **Conf. Inter. 95%** | [-7.58 to 39.54] |  | **Conf. Inter. 95%** | [-0.38 to 5.37] |
|  | **Population mean post-constraints** | Reduced |  | **Population mean post-constraints** | Reduced |
| **V _20Gy_** | **MWU test result** | NOT Sig diff. | **D_max_** | **MWU test result** | NOT Sig diff. |
|  | **P-Value** | 0.108 |  | **P-Value** | 0.108 |
|  | **Mean Diff.** | 13.37 |  | **Mean Diff.** | -2.22 |
|  | **Conf. Inter. 95%** | [-6.91 to 33.66] |  | **Conf. Inter. 95%** | [-5.95 to 1.52] |
|  | **Population mean post-constraints** | Reduced |  | **Population mean post-constraints** | Increased |

Table 11 - P-values and mean difference at 95% confidence interval for the trachea at all dosimetric points evaluated in this study.

| **Trachea** | | | | | |
| --- | --- | --- | --- | --- | --- |
| **Dosimetric parameter** | **Evaluation** | **Result** | **Dosimetric parameter** | **Evaluation** | **Result** |
| **V _5Gy_** | **MWU test result** | No Change | **D_mean_** | **MWU test result** | NOT Sig diff. |
|  | **P-Value** | No p-value |  | **P-Value** | 0.500 |
|  | **Mean Diff.** | - |  | **Mean Diff.** | 0.91 |
|  | **Conf. Inter. 95%** | - |  | **Conf. Inter. 95%** | [-3.50 to 5.31] |
|  | **Population mean post-constraints** | Equal |  | **Population mean post-constraints** | Reduced |
| **V _10Gy_** | **MWU test result** | NOT Sig diff. | **D_median_** | **MWU test result** | NOT Sig diff. |
|  | **P-Value** | 0.500 |  | **P-Value** | 0.500 |
|  | **Mean Diff.** | 1.15 |  | **Mean Diff.** | 1.01 |
|  | **Conf. Inter. 95%** | [-6.95 to 9.26] |  | **Conf. Inter. 95%** | [-3.41 to 5.42] |
|  | **Population mean post-constraints** | Reduced |  | **Population mean post-constraints** | Reduced |
| **V _15Gy_** | **MWU test result** | NOT Sig diff. | **D_min_** | **MWU test result** | NOT Sig diff. |
|  | **P-Value** | 0.500 |  | **P-Value** | 0.305 |
|  | **Mean Diff.** | 1.29 |  | **Mean Diff.** | 0.76 |
|  | **Conf. Inter. 95%** | [-23.08 to 25.66] |  | **Conf. Inter. 95%** | [-2.72 to 4.24] |
|  | **Population mean post-constraints** | Reduced |  | **Population mean post-constraints** | Reduced |
| **V _20Gy_** | **MWU test result** | NOT Sig diff. | **D_max_** | **MWU test result** | NOT Sig diff. |
|  | **P-Value** | 0.449 |  | **P-Value** | 0.351 |
|  | **Mean Diff.** | 6.79 |  | **Mean Diff.** | -0.62 |
|  | **Conf. Inter. 95%** | [-28.20 to 41.78] |  | **Conf. Inter. 95%** | [-5.64 to 4.40] |
|  | **Population mean post-constraints** | Reduced |  | **Population mean post-constraints** | Increased |

Table 12 - P-values and mean difference at 95% confidence interval for the thyroid at all dosimetric points evaluated in this study.

| **Thyroid** | | | | | |
| --- | --- | --- | --- | --- | --- |
| **Dosimetric parameter** | **Evaluation** | **Result** | **Dosimetric parameter** | **Evaluation** | **Result** |
| **V _5Gy_** | **MWU test result** | No Change | **D_mean_** | **MWU test result** | NOT Sig diff. |
|  | **P-Value** | No p-value |  | **P-Value** | 0.318 |
|  | **Mean Diff.** | - |  | **Mean Diff.** | -0.97 |
|  | **Conf. Inter. 95%** | - |  | **Conf. Inter. 95%** | [-4.86 to 2.92] |
|  | **Population mean post-constraints** | Equal |  | **Population mean post-constraints** | Increased |
| **V _10Gy_** | **MWU test result** | NOT Sig diff. | **D_median_** | **MWU test result** | NOT Sig diff. |
|  | **P-Value** | 0.316 |  | **P-Value** | 0.282 |
|  | **Mean Diff.** | -8.51 |  | **Mean Diff.** | -1.17 |
|  | **Conf. Inter. 95%** | [-23.90 to 6.88] |  | **Conf. Inter. 95%** | [-5.15 to 2.81] |
|  | **Population mean post-constraints** | Increased |  | **Population mean post-constraints** | Increased |
| **V _15Gy_** | **MWU test result** | NOT Sig diff. | **D_min_** | **MWU test result** | NOT Sig diff. |
|  | **P-Value** | 0.282 |  | **P-Value** | 0.396 |
|  | **Mean Diff.** | -10.16 |  | **Mean Diff.** | 0.19 |
|  | **Conf. Inter. 95%** | [-46.12 to 25.81] |  | **Conf. Inter. 95%** | [-2.64 to 3.02] |
|  | **Population mean post-constraints** | Increased |  | **Population mean post-constraints** | Reduced |
| **V _20Gy_** | **MWUU test result** | NOT Sig diff. | **D_max_** | **MWU test result** | NOT Sig diff. |
|  | **P-Value** | 0.135 |  | **P-Value** | 0.318 |
|  | **Mean Diff.** | -3.94 |  | **Mean Diff.** | -0.58 |
|  | **Conf. Inter. 95%** | [-30.60 to 22.73] |  | **Conf. Inter. 95%** | [-5.93 to 4.77] |
|  | **Population mean post-constraints** | Increased |  | **Population mean post-constraints** | Increased |
